# Evaluation of the protective effect of losartan in acetaminophen-induced liver and kidney damage in mice

**DOI:** 10.1007/s00210-023-02937-0

**Published:** 2024-01-09

**Authors:** Serkan Şahin, Ayça Çakmak Aydın, Ayşe Yeşim Göçmen, Emin Kaymak

**Affiliations:** 1 Department of Medical Pharmacology, Faculty of Medicine, Yozgat Bozok University, Yozgat, Turkey; 2 Department of Biochemistry, Faculty of Medicine, Yozgat Bozok University, Yozgat, Turkey; 3 Department of Histology, Faculty of Medicine, Yozgat Bozok University, Yozgat, Turkey

**Keywords:** Acetaminophen, Losartan, GW9662, Hepatotoxicity, Nephrotoxicity

## Abstract

Acetaminophen is widely used among humans as an antipyretic and analgesic. In this study, the protective effect of losartan in hepatotoxicity and nephrotoxicity induced by acetaminophen in mice was investigated owing to its anti-inflammatory and antioxidant effects. An injection of a single dose of 500 mg/kg (i.p.) acetaminophen was administered to induce hepatotoxicity and nephrotoxicity in Groups VI–X. Losartan at doses of 1 mg/kg (Group VII), 3 mg/kg (Group VIII), and 10 mg/kg (Groups III, V, IX, and X) was injected intraperitoneally twice, at 1 and 12 h after the acetaminophen injection. Additionally, a 4 mg/kg dose of GW9662 (peroxisome proliferator-activated receptor gamma (PPAR-γ) antagonist) was injected intraperitoneally 30 min before the losartan injections in Groups V and X. At the end of 24 h, the mice were euthanized, and blood, liver, and kidney tissue samples were collected. Levels of AST, ALT, creatinine, and oxidative stress markers including TBARS, SOD, CAT, GPx, TAS, TOS, GSH, and GSSG, along with pro-inflammatory cytokines IL-1β, IL-6, IL-8, IL-10, IL-17, and TNF-α, were measured using ELISA kits. Additionally, a histological evaluation of the tissue samples was performed. Acetaminophen causes increases in the levels of AST, ALT, creatinine, TBARS, TOS, GSSG, IL-1β, IL-6, IL-8, IL-10, IL-17, and TNF-α in serum, liver, and kidney tissue. Meanwhile, it led to a decrease in the levels of SOD, CAT, GPx, TAS, and GSH. Losartan injection reversed oxidative and inflammatory damage induced by acetaminophen. Histopathological changes in liver and kidney tissue were alleviated by losartan. The substance GW9662 increased the protective effect of losartan. In light of all the data obtained from our study, it can be said that losartan has a protective effect on liver and kidney damage induced by acetaminophen due to its antioxidant and anti-inflammatory effects. In terms of the study, losartan was found to be an alternative substance that could protect people from the harmful effects of acetaminophen.

## Introduction

The liver plays a very important role in the metabolism of drugs and other xenobiotics. Liver damage may occur as a result of excessive intake of drugs and other xenobiotics (Al Kury et al. 2020; Abbasi et al. 2021). Excessive augmentation in the levels of free oxygen radicals causes a decline of thiols and consequently lipid peroxidation. Lipid peroxidation can cause cell membrane damage and then liver damage (Al Kury et al. 2020).

Acetaminophen is one of the most used non-prescription drugs for antipyretic-analgesic purposes in most countries. For this reason, acetaminophen is one of the drugs that most commonly cause predictable liver damage (Budnitz et al. 2011). Liver damage caused by drugs is a great problem worldwide. Drug-induced liver damage can be divided into two classes, idiosyncratic and predictable liver damage (Hartmut et al. 2014). Idiosyncratic liver damage can occur mainly in susceptible patients, at therapeutic doses, days or months after initiation of therapy. Although it is estimated that adaptive immune mechanisms play a substantial role in the emergence of idiosyncratic liver damage, the exact mechanism of occurrence has not been fully defined (Kaplowitz 2005; Uetrecht and Naisbitt 2013). Predictable liver damage is mainly caused by deliberate or accidental drug overdose.

Acetaminophen causes its harmful effects on the liver mainly through N-acetyl-p-benzoquinoneimine, an extremely toxic metabolite formed by cytochrome CYP 2E1 and CYP 1A2 enzymes. This metabolite can bind covalently to some intracellular molecules such as DNA, reduce glutathione (GSH), bring about oxidative stress, and change the amount of calcium and/or thiol in hepatocytes. As a result of these events, liver failure may develop (Zaher et al. 1998; Sohrabinezhad et al. 2019). However, hepatic inflammatory cytokines have a role in liver damage caused by acetaminophen (Al Humayed et al. 2019).

Acetaminophen-induced hepatotoxicity in mice resembles hepatotoxicity in humans taking overdoses of acetaminophen in many basic mechanisms. However, liver damage after an overdose of acetaminophen develops more rapidly in mice than in humans. The mouse model of acetaminophen-induced hepatotoxicity was described in the 1970s (Mitchell et al. 1973). Alanine aminotransferase (ALT), one of the indicators of liver cell death, reaches its highest level between 12 and 24 h in mice and 36–48 h in humans after a taking overdose of acetaminophen (Larson 2007; McGill et al. 2013). Although nephrotoxicity due to acetaminophen overdose is less common than hepatotoxicity, it can cause single or multiple organ failure. Renal failure has been reported in 1–2% of patients exposed to acetaminophen overdose (Güvenç et al. 2020).

PPAR-γ has been demonstrated to have a protective effect on tissue protection and repair, especially in ischemic damage (Koh et al. 2013). PPAR-γ activation inhibits inflammation and oxidative stress. Angiotensin receptor blockers (ARBs) have been shown to have an agonistic effect on PPAR-γ. ARBs also reduce oxidative stress (Goyal et al. 2011; Pang et al. 2012; Helal and Samra 2020).

Losartan (2-N-butyl-4-chloro-5-hydroxymethyl-1-[(2′-(1H-tetrazol-5-yl)biphenyl-4-yl)methyl]imidazole) is a genuine angiotensin II (Ang II) type I receptor (AT1R) inhibitör. It is used for the balance of fluid homeostasis and blood pressure. Losartan has been shown to have a protective effect on the liver in different models of nephrotoxicity and hepatotoxicity. However, Ang II enhances vascular permeability, stimulates inflammatory cells, and activates proinflammatory cytokines and chemokines. Losartan has been shown to exert a protective effect through PPAR-γ in various experimental models of ischemia/reperfusion (Koh et al. 2013).

Our study is the first to assess the impact of losartan on liver and kidney damage induced by acetaminophen. This study aimed to investigate the availability of an alternative substance that can protect people from these harmful effects of acetaminophen, which is widely used by people as an analgesic–antipyretic and causes hepato-renal problems at a high rate.

## Materials and methods

### Chemicals

Acetaminophen, Losartan, and GW9662 were purchased from Boston USA Chemistry, MA, USA.

### Animals

In this study, approval was obtained from the Erciyes University Animal Experiments Local Ethics Committee (approval decision no: 20/112). A total of 130 male and female balb/c mice (weight, 25–30 gr.) were obtained from Erciyes University Experimental Research Application and Research Center. In the environment where animals are present (22 °C ± 2 °C), 12 h of light/12 h of dark lighting cycle and an average humidity of 50 ± 5% were provided. The animals’ water and feed intake are freed (Helal and Samra 2020). In order for the animals to get used to the experimental environment, they were started to be kept in the environment where the experiments were carried out one week in advance.

### Experimental design

Feeding to mice was stopped 10 h before the start of the experiments, but water was continued. Mice were divided into 10 groups with 13 mice in each group. Hepatotoxicity was not established in the first 5 groups, which were the control groups. In the VI and X groups, hepatotoxicity and nephrotoxicity were induced by intraperitoneal administration of 500 mg/kg acetaminophen (Kaushik et al. 2017a; Zhao et al. 2018). The 10 groups are as follows:

- Group I (CTRL) (*n*:13); 0.9% saline was injected (i.p.) (Helal and Samra 2020).
- Group II (CTRL/DMSO) (*n*:13); 4% DMSO was injected (i.p.) (6,7,13).
- Group III (CTRL/LOS10) (*n*:13); Losartan was injected (i.p.) at a dose of 10 mg/kg 1 h and 12 h after the start of the experiments (Zhang et al. 2012; Koh et al. 2013).
- Group IV (CTRL/GW9662) (*n*:13); 30 min and 11.5 h after the start of the experiments, GW9662, a PPAR-γ antagonist at a dose of 4 mg/kg, was injected (i.p.) (dissolved in 4% DMSO) (Zhang et al. 2012; Koh et al. 2013; Helal and Samra 2020).
- Group V (CTRL/LOS10/GW9662) (*n*:13); Losartan was injected (i.p.) at a dose of 10 mg/kg 1 h and 12 h after the start of the experiments (Zhang et al. 2012; Koh et al. 2013). GW9662, a PPAR-γ antagonist was injected (i.p.) at a dose of 4 mg/kg 30 min prior to losartan injections, (dissolved in 4% DMSO) (Zhang et al. 2012; Koh et al. 2013; Helal and Samra 2020).
- Group VI (APAP) (*n*:13); Acetaminophen was injected at a dose of 500 mg/kg (i.p.) (Helal and Samra 2020).
- Group VII (APAP/LOS1) (*n*:13); 1 mg/kg losartan was injected (i.p.) twice, 1 h and 12 h after the acetaminophen injection (Zhang et al. 2012; Koh et al. 2013; Helal and Samra 2020).
- Group VIII (APAP/LOS3) (*n*:13); 3 mg/kg losartan was injected (i.p.) twice, 1 h and 12 h after the acetaminophen injection (Zhang et al. 2012; Koh et al. 2013; Helal and Samra 2020).
- Group IX (APAP/LOS10) (*n*:13); 10 mg/kg losartan was injected (i.p.) twice, 1 h and 12 h after the acetaminophen injection (Zhang et al. 2012; Koh et al. 2013; Helal and Samra 2020).
- Group X (APAP/LOS10/GW9662) (*n*:13); 10 mg/kg losartan was injected (i.p.) twice, 1 h and 12 h after the acetaminophen injection. In addition, GW9662, a PPAR-γ antagonist was injected (i.p.) at a dose of 4 mg/kg 30 min prior to losartan injections, (dissolved in 4% DMSO) (Zhang et al. 2012; Koh et al. 2013; Helal and Samra 2020).

The dose of acetaminophen, losartan, and GW9662 was determined as a result of the literature review (Zhang et al. 2012; Koh et al. 2013; Helal and Samra 2020). At 24 h after acetaminophen injection, animals were sacrificed under ketamine (75 mg/kg)/xylazine (10 mg/kg) anesthesia, and blood samples were obtained. Blood samples were centrifuged at 3000 rpm for 5 min, serum-separated, and stored at −80 °C. The liver and kidneys of the animals were taken and sectioned into two pieces. One of the pieces was fixed in 10% formaldehyde for histopathological examinations. The other piece was used for biochemical analysis. Samples taken for biochemical analysis were stored at −80 °C until analysis (Ulusoy et al. 2016; Abdel-Daim et al. 2017; Kalantari et al. 2019; Helal and Samra 2020).

### Determination of serum transaminases

Serum levels of hepatic function markers alanine aminotransferase (ALT), aspartate aminotransferase (AST), and creatinine were determined using commercial high-quality optimized Elabscience® ELISA test kits (MD, USA) in a microplate reader, according to the manufacturer’s instructions (Zhao et al. 2018; Helal and Samra 2020).

### Determination of antioxidant indices and proinflammatory cytokines

Superoxide dismutase (SOD), catalase (CAT), and glutathione peroxidase (GPx) activities and Glutathione (GSH) and oxidized glutathione (GSSG) levels were measured in blood and tissue samples by modified methods (El-Sokkary et al. 2007). Colorimetric kits were used to measure the total oxidant state (TOS) and total antioxidant state (TAS), and malondialdehyde (MDA) was measured as thiobarbituric acid reactive substances (TBARS). The analyses were repeated twice for each sample. Glutathione content was calculated using the formula GSH = Total − GSH(T − GSH) − (2 × GSSG). The results of GSH and GSSG were normalized to the total protein content and were expressed as nmol of GSH or GSSG per mg of protein (nmol GSH/mg protein or nmol GSSG/mg protein). Oxidative stress markers TAS and TOS levels were analyzed as previously described (Yılmaz et al. 2020). Commercial enzyme-linked immunosorbent (ELISA) assay kits were used to measure the serum levels of cytokines (interleukin (IL) −1β, −6, −8, −10, −17 and tumor necrosis factor-α (TNF-α) (Elabscience, MD, USA)).

### Histopathological examination of liver and kidney tissue

At the end of the experiment, liver and kidney tissues were fixed in 10% formaldehyde solution. After fixation, the tissues were dehydrated by passing through a series of increasing grades of alcohol (50%, 70%, 80%, 96%, 100%). Tissues cleared with xylene were embedded in paraffin. Hematoxylin–Eosin (H+E) staining was applied to 5–6-µm thick sections taken from paraffin blocks, covered with closure solution (Entellan®, Merck), and examined under Olimpus BX3 microscope (Zhao et al. 2018; Akin et al. 2021; Kaymak et al. 2022).

### Statistical analysis

Results are presented as mean ± standard error. Data analysis IBM SPSS 23.0 package program was used. The distribution characteristics of the data were determined by the Kolmogorow–Smirnov test. One-way analysis of variance was used for normally distributed datum, followed by Tukey’s post hoc test. One-way analysis of variance was used for normally distributed data, followed by Tukey’s post hoc test. *P* < 0.05 was considered statistically significant.

## Results

A total of 130 male and female balb/c mice were included in the study. During the experiment, there was no death in both control and acetaminophen-induced hepatoxicity and nephrotoxicity groups. All the data obtained as a result of the study are shown in the tables. 

### Effect of losartan on serum parameters in acetaminophen toxicity

In the APAP group, compared to the CTRL group, there was an increase in ALT, AST, creatinine, IL-1β, IL-6, IL-8, IL-10, IL-17, TNF-α, TOS, GSSG, and MDA levels by 8.5, 7.5, 7.3, 7.1, 6.1, 4.1, 3.6, 1.6, 3.2, 3.7, 3.8, and 7.5 times, respectively. TAS and GSH levels were decreased by 2.2 and 2.7 times. A dose-dependent decline in AST, ALT, Creatinine, IL-1β, IL-6, IL-8, IL-10, IL-17, TNF-α, TOS, GSSG, and MDA levels was observed in the LOS1, LOS3, and LOS10 groups after acetaminophen administration. In the APAP/LOS10/GW9662 group, a decrease was detected in ALT, AST, creatinine, IL-1β, IL-6, IL-8, IL-10, IL-17, TNF-α, TOS, GSSG, and MDA levels (Table 1).

**Table 1:** Effect of losartan on serum biochemical values in acetaminophen toxicity

Serum groups	CTRL	CTRL/DMSO	CTRL/LOS10	CTRL/GW9662	CTRL/LOS10/GW9662	APAP	APAP/LOS1	APAP/LOS3	APAP/LOS10	APAP/LOS10/GW9662
ALT (U/L)	^$^46.5 ± 1	^$^64.6 ± 3.3	^$^58.9 ± 2.1	^$^45.5 ± 0.7	^$^48.3 ± 0.9	^#^395.4 ± 8.4	^#$^226.8 ± 3.1	^#$^189.1 ± 0.3	^#$^123.4 ± 0.2	^#$^85.7 ± 0.1
AST (U/L)	^$^101.7 ± 1.8	^$^115.4 ± 4	^$^118.8 ± 4.8	^$^120.8 ± 6	^$^191.2 ± 50.7	^#^765.7 ± 16.7	^#$^372.3 ± 43.5	^#$^242.2 ± 0.3	^#$^166.7 ± 0.2	^#$^123.1 ± 0.1
Creatinine (mg/dl)	^$^4.1 ± 0.1	^$^5.4 ± 0.2	^$^5.1 ± 0.2	^$^4.8 ± 0.1	^$^5.1 ± 0.1	^#^30.2 ± 0.8	^#$^22.1 ± 0.3	^#$^18.7 ± 0.02	^#$^12 ± 0.04	^#$^8.5 ± 0.01
IL-1β (pg/ml)	^$^8.4 ± 0.2	^$^10.9 ± 0.4	^$^10.2 ± 0.3	^$^8.6 ± 0.05	^$^9.3 ± 0.1	^#^59.1 ± 1.6	^#$^41.8 ± 0.6	^#$^34.2 ± 0.05	^#$^24.3 ± 0.1	^#$^16.1 ± 0.1
IL-6 (pg/ml)	^$^44.4 ± 1.6	^$^56.3 ± 1.9	^$^48.7 ± 1.7	^$^36.5 ± 0.5	^$^39.7 ± 4	^#^271.4 ± 6.7	^#$^200.3 ± 2.6	^#$^171.3 ± 1.7	^#$^114.7 ± 1.3	^#$^80 ± 0.8
IL-8 (pg/ml)	^$^117 ± 2.5	^$^136.1 ± 3.1	^$^124 ± 2.7	^$^104.4 ± 0.8	^$^109.5 ± 6.4	^#^480.3 ± 10.7	^#$^366.5 ± 4.2	^#$^320.1 ± 2.8	^#$^229.5 ± 2	^#$^174 ± 1.3
IL-10 (pg/ml)	^$^171.3 ± 3.2	^$^195.1 ± 3.9	^$^180 ± 3.3	^$^155.5 ± 1	^$^161.9 ± 8.1	^#^625.4 ± 13.4	^#$^483.2 ± 5.3	^#$^425.1 ± 3.5	^#$^311.9 ± 2.5	^#$^242.5 ± 1.6
IL-17 (pg/ml)	^$^32.4 ± 1.9	^$^33.3 ± 1.5	^$^28.1 ± 1	^#$^9.9 ± 2.3	^#$^17.8 ± 5.8	^#^50.8 ± 1.2	^#$^14.3 ± 5	^#$^6.9 ± 0.3	^#$^5.3 ± 0.2	^$^4.7 ± 0.2
TNF-α (pg/ml)	^$^225.6 ± 7.2	^$^236.6 ± 6.2	^$^218.2 ± 5.9	^$^195.7 ± 1.2	^$^209.9 ± 11	^#^713.4 ± 17.2	^#$^507.4 ± 15	^#$^425.1 ± 3.5	^#$^311.9 ± 2.5	^#$^242.5 ± 1.6
TAS (mmol/L)	^$^7.2 ± 0.2	^#$^9.3 ± 0.3	^#$^8.6 ± 0.3	^$^7.2 ± 0.04	^$^7.7 ± 0.1	^#^3.3 ± 0.1	^#^3.7 ± 0.1	^#^3.5 ± 0.04	^#$^4.2 ± 0	^#$^6.5 ± 0.02
TOS (µmol/L)	^$^32 ± 0.4	^$^36.5 ± 0.7	^$^34.8 ± 0.6	^$^32.8 ± 0.2	^$^33.9 ± 0.3	^#^119 ± 2.8	^#$^90.4 ± 1	^#$^78 ± 0.2	^#$^56.8 ± 0.1	^#$^44.9 ± 0.1
GSH (µmol/L)	^$^3.3 ± 0.1	^#$^3.8 ± 0.2	^$^3.5 ± 0.1	^#$^2.9 ± 0.02	^$^3 ± 0.04	^#^1.2 ± 0.03	^#^1.2 ± 0.02	^#^1.2 ± 0.002	^#^1.5 ± 0.002	^#$^2 ± 0.002
GSSG (µmol/L)	^$^0.3 ± 0.002	^$^0.3 ± 0.01	^$^0.3 ± 0.005	^$^0.3 ± 0.001	^$^0.3 ± 0.002	^#^1 ± 0.02	^#$^0.7 ± 0.01	^#$^0.6 ± 0.001	^#$^0.5 ± 0.0003	^#$^0.4 ± 0.0002
MDA (nmol/ml)	^$^1.1 ± 0.02	^$^1.5 ± 0.1	^$^1.4 ± 0.05	^$^1.1 ± 0.01	^$^1.2 ± 0.02	^#^8.3 ± 0.2	^#$^5.8 ± 0.1	^#$^4.8 ± 0.01	^#$^3.1 ± 0.003	^#$^2.2 ± 0.002

All values were expressed as mean ± standard error

*CTRL* control, *APAP* acetaminophen, *LOS* losartan, *GW9662* peroxisome proliferator-activated receptor gamma (PPARγ) antagonist, *ALT* alanine aminotransferase, *AST* aspartate aminotransferase, *TNF-α* tumor necrosis factor, *IL-1β* ınterleukin-1β, *IL-6* ınterleukin-6, *IL-8* ınterleukin-8, *IL-10* ınterleukin-10, *IL-17* ınterleukin-17, *TOS* total oxidant level, *TAS* total antioxidant level, *MDA* malondialdehyde, *GSH* glutathione, *GSSG* oxidized glutathione

^$^Shows the group that is significantly different from the APAP group (*p* < 0.05)

^#^Shows the group that is significantly different from the CTRL group (*p* < 0.05)

### Effect of losartan on liver parameters in acetaminophen toxicity

It was observed that TBARS, TOS, and GSSG values were increased in the APAP group compared to the CTRL group (*p* < 0.05). It was determined that this increase is 1.6, 1.1, and 1.8 times, respectively. SOD, CAT, GPx, TAS, and GSH values were decreased in the APAP group compared to the CTRL group. This decrease is 1.3, 1.5, 1.7, 1.3, and 1.2 times, respectively. A statistically significant increase in GPx, TAS, and GSH values was observed in the APAP/LOS1, APAP/LOS3, APAP/LOS10, and APAP/LOS10/GW9662 groups compared to the APAP group (*p* < 0.05). A decrease in TBARS, TOS, and GSSG values was observed in the APAP/LOS1, APAP/LOS3, APAP/LOS10, and APAP/LOS10/GW9662 groups compared to the APAP group (Table 2).

**Table 2:** The effect of losartan on liver biochemical values in acetaminophen toxicity

Groups’ liver	CTRL	CTRL/ DMSO	CTRL/LOS10	CTRL/GW9662	CTRL/LOS10/GW9662	APAP	APAP/LOS1	APAP/LOS3	APAP/LOS10	APAP/LOS10/GW9662
TBARS (nmol MDA/g protein)	^$^3.2 ± 0.03	^$^3.1 ± 0.1	^$^3.3 ± 0.1	^$^3.1 ± 0.02	^$^2.9 ± 0.1	^#^5.3 ± 0.6	^#$^7.3 ± 0.1	^#^5.7 ± 0.1	^#^5.3 ± 0.03	^#^4.6 ± 0.03
SOD (U/mg protein)	^$^19.6 ± 0.2	17.6 ± 0.9	17.1 ± 1	^$^19.8 ± 0.1	17.8 ± 1	^#^15.2 ± 1	17.4 ± 0.2	^#^15.4 ± 0.9	^#^14.9 ± 1	^#^15 ± 1
CAT (U/mg protein)	^$^28.9 ± 1.1	^$^26.7 ± 1.4	^$^24 ± 0.1	^$^25.8 ± 0.1	^$^26 ± 1.1	^#^19.2 ± 1.1	^#^19.6 ± 0.1	^#^19.8 ± 1.1	^#^19.6 ± 1.4	^#^22.3 ± 1
GPx (U/mg protein)	^$^68.9 ± 0.5	^$^69.1 ± 2.6	^$^67.2 ± 2.6	^$^68.9 ± 0.4	^$^63.9 ± 1.7	^#^40.8 ± 1.7	^#^41.2 ± 0.3	^#$^50.4 ± 2.4	^#$^49.7 ± 2.3	^#$^52 ± 1.6
TAS (mmol trolox Equiv/mg prt)	^$^2.3 ± 0.002	^$^2.3 ± 0.004	^$^2.3 ± 0.003	^$^2.3 ± 0.001	^$^2.2 ± 0.04	^#^1.8 ± 0.003	^#$^1.8 ± 0.0004	^#$^1.9 ± 0.01	^#$^1.9 ± 0.02	^#$^2 ± 0.003
TOS (μmol H2O2 Equiv/mg prt)	6.6 ± 0.5	6.2 ± 0.4	7.3 ± 0.4	^#$^4.5 ± 0.2	^#$^4.3 ± 0.1	7.4 ± 0.6	^#$^10.5 ± 0.2	^#^8.4 ± 0.1	7.5 ± 0.6	7 ± 0.4
GSH (nmol/mg prt)	^$^8.9 ± 0.02	^$^8.9 ± 0.04	^$^8.9 ± 0.03	^$^8.9 ± 0.01	^$^8.7 ± 0.1	^#^7.2 ± 0.03	^#^7.2 ± 0.01	^#$^7.6 ± 0.04	^#$^7.7 ± 0.03	^#$^8.1 ± 0.02
GSSG (nmol/mg prt)	^$^2.1 ± 0.1	^$^2.1 ± 0.1	^$^2.1 ± 0.1	^$^1.9 ± 0.03	^$^1.9 ± 0.04	^#^3.9 ± 0.8	^#$^6.1 ± 0.1	^#^4.7 ± 0.04	^#^4 ± 0.2	3.2 ± 0.2

All values were expressed as mean ± standard error

*CTRL* control, *APAP* acetaminophen, *LOS* losartan, *GW9662* peroxisome proliferator-activated receptor gamma (PPARγ) antagonist, *TBARS* thiobarbituric acid reactive substances, *CAT* catalase, *GPx* glutathione peroxidase, *SOD* superoxide dismutase, *TOS* total oxidant level, *TAS* total antioxidant level, *GSH* glutathione, *GSSG* oxidized glutathione

^$^Shows the group that is significantly different from the APAP group (*p* < 0.05)

^#^Shows the group that is significantly different from the CTRL group (*p* < 0.05)

### Effect of losartan on renal parameters in acetaminophen toxicity

An increase in TBARS, TOS, and GSSG values was observed in the APAP group compared to the CTRL group (*p* < 0.05). It was determined this increase is 2.7, 2.5, and 3.1 times, respectively. SOD, CAT, GPx, TAS, and GSH values were decreased in the APAP group compared to the CTRL group. This decrease is 1.2, 1,2, 1.5, 1.3, and 1.8 times, respectively. A decrease was observed in TOS and GSSG values in the APAP/LOS3 (*p* > 0.05), APAP/LOS10 (*p* < 0.05), and APAP/LOS10/GW9662 (*p* < 0.05) groups compared to the APAP group (Table 3).

**Table 3:** The effect of losartan on kidney biochemical values in acetaminophen toxicity

Groups’ kidney	CTRL	CTRL/DMSO	CTRL/LOS10	CTRL/GW9662	CTRL/LOS10/GW9662	APAP	APAP/LOS1	APAP/LOS3	APAP/LOS10	APAP/LOS10/GW9662
TBARS (nmol MDA/g protein)	^$^1.2 ± 0.03	^$^1.2 ± 0.1	^$^1.3 ± 0.1	^$^1.3 ± 0.02	^$^1.3 ± 0.03	^#^3.2 ± 0.6	^#$^5.7 ± 0.1	^#$^4.3 ± 0.02	^#^3.3 ± 0.02	^#^2.6 ± 0.02
SOD (U/mg protein)	18.3 ± 1	15.1 ± 0.6	15.6 ± 0.2	^$^19.8 ± 0.1	17.8 ± 1	15.2 ± 1	17.4 ± 0.2	15.4 ± 0.9	^#^14.9 ± 1	^#^15 ± 1
CAT (U/mg protein)	20.2 ± 0.1	^$^20.5 ± 1.2	19.6 ± 1.1	20.3 ± 0.1	^$^20.6 ± 1.2	16.2 ± 1.1	16.6 ± 0.1	16.8 ± 1.1	16 ± 1.1	16.6 ± 1.1
GPx (U/mg protein)	^$^58.5 ± 1.2	^$^59.7 ± 1.6	^$^59.5 ± 1.1	^#$^68.9 ± 0.4	^$^62.2 ± 2.5	^#^38.8 ± 1	^#^41.2 ± 0.3	^#$^48.2 ± 1.8	^#$^47.6 ± 1.4	^#$^51.5 ± 1.4
TAS (mmol trolox equiv/mg prt)	^$^2.3 ± 0.001	^$^2.3 ± 0.002	^$^2.3 ± 0.003	^$^2.3 ± 0.001	^#$^2.2 ± 0.04	^#^1.8 ± 0.003	^#^1.8 ± 0.0004	^#$^1.9 ± 0.01	^#$^1.9 ± 0.02	^#$^2 ± 0.003
TOS (µmol H2O2 equiv/mg prt)	3.9 ± 0.1	^$^4.4 ± 0.1	^$^4.2 ± 0.1	^$^4.1 ± 0.04	^$^4.2 ± 0.02	^#^10 ± 0.8	^#^11.2 ± 0.2	^#^9 ± 0.02	^#$^6.7 ± 0.01	^#$^5.2 ± 0.01
GSH (nmol/mg prt)	^$^6.1 ± 0.01	^$^6.2 ± 0.01	^$^6.1 ± 0.01	^$^6.1 ± 0.003	^#$^5.7 ± 0.3	^#^3.5 ± 0.01	^#^3.4 ± 0.001	^#$^4.2 ± 0.1	^#$^4.4 ± 0.05	^#$^4.7 ± 0.01
GSSG (nmol/mg prt)	^$^1.4 ± 0.03	^$^1.5 ± 0.04	^$^1.5 ± 0.1	^$^1.4 ± 0.01	^$^1.5 ± 0.02	^#^4.3 ± 0.5	^#$^5 ± 0.1	^#^4 ± 0.02	^#$^3.1 ± 0.1	^#$^2.4 ± 0.1

All values were expressed as mean ± standard error

*CTRL* control, *APAP* acetaminophen, *LOS* losartan, *GW9662* peroxisome proliferator-activated receptor gamma (PPARγ) antagonist, *TBARS* thiobarbituric acid reactive substances, *SOD* superoxide dismutase, *CAT* catalase, *GPx* glutathione peroxidase, *TAS* total antioxidant level, *TOS* total oxidant level, *GSH* glutathione, *GSSG* oxidized glutathione

^$^Shows the group that is significantly different from the APAP group (*p* < 0.05)

^#^Shows the group that is significantly different from the CTRL group (*p* < 0.05)

### Effect of losartan on liver histopathology in acetaminophen toxicity

**Fig. 1:**
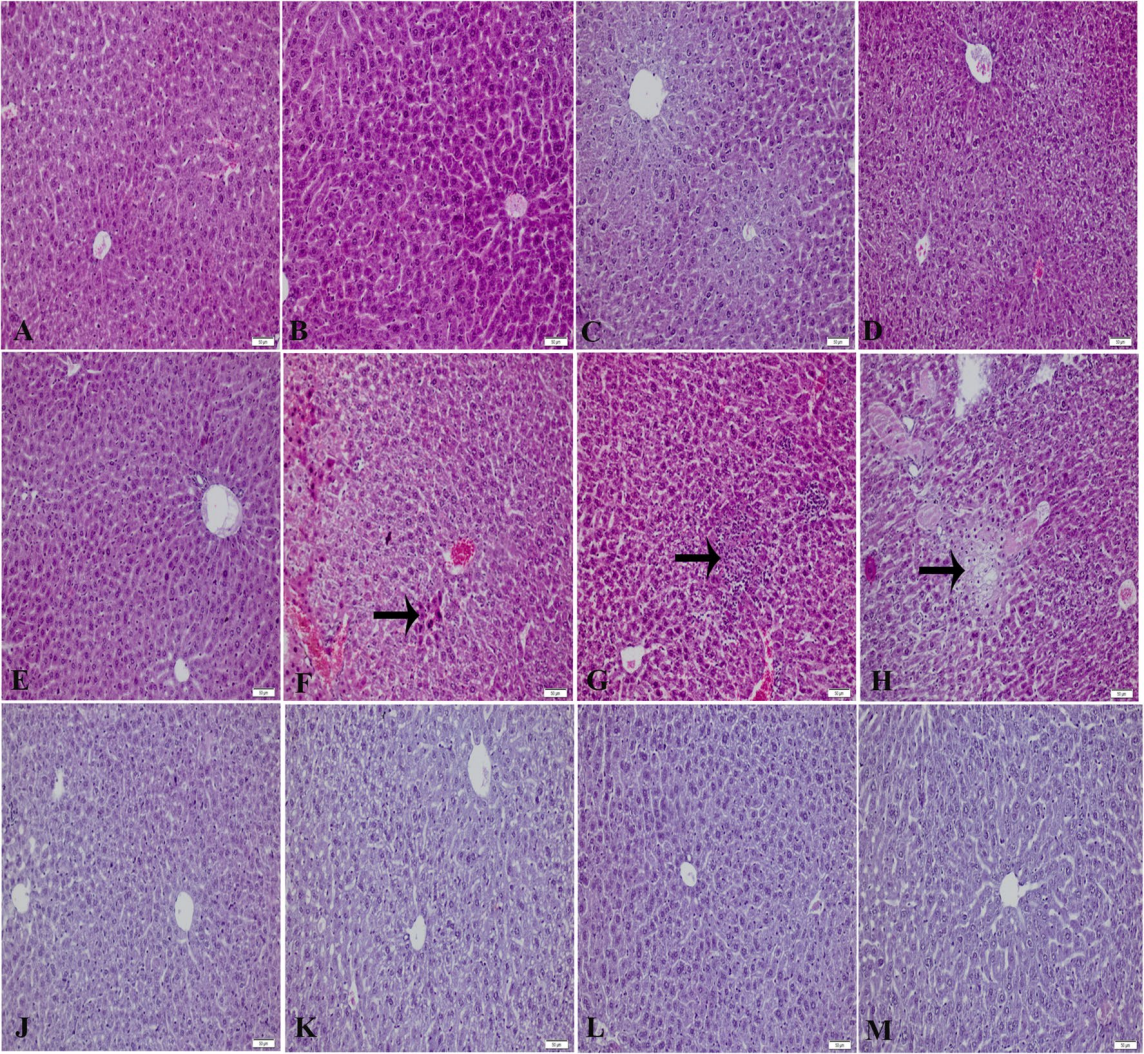
Liver tissue hematoxylin and eosin images. **A** CTRL group; **B** CTRL/DMSO group; **C** CTRL/LOS10 group; **D** CTRL/GW9662 group; **E** CTRL/LOS10/GW9662 group; **F**, **G**, and **H** APAP group (the arrow shows necrotic cells in **F**, the arrow shows inflammatory areas in **G**, the arrow shows cells with pycnotic nuclei in **H**); **J** APAP/LOS1 group; **K** APAP/LOS3 group; **L** APAP/LOS10 group; and **M** indicates APAP/LOS10/GW9662 group. Image magnification at 200×

Liver tissue staining images are shown in Fig. 1. Normal histological images were obtained in the liver tissues of the CTRL, CTRL/DMSO, CTRL/LOS10, CTRL/GW9662, and CTRL/LOS10/GW9662 groups. Necrotic cells, pycnotic nuclei, inflammatory areas, and vacuolized hepatocytes were observed in the liver tissue of the APAP group. These damages were absent in the APAP/LOS1, APAP/LOS3, APAP/LOS10, and APAP/LOS10/GW9662 groups.

### Effect of losartan on renal histopathology in acetaminophen toxicity

**Fig. 2:**
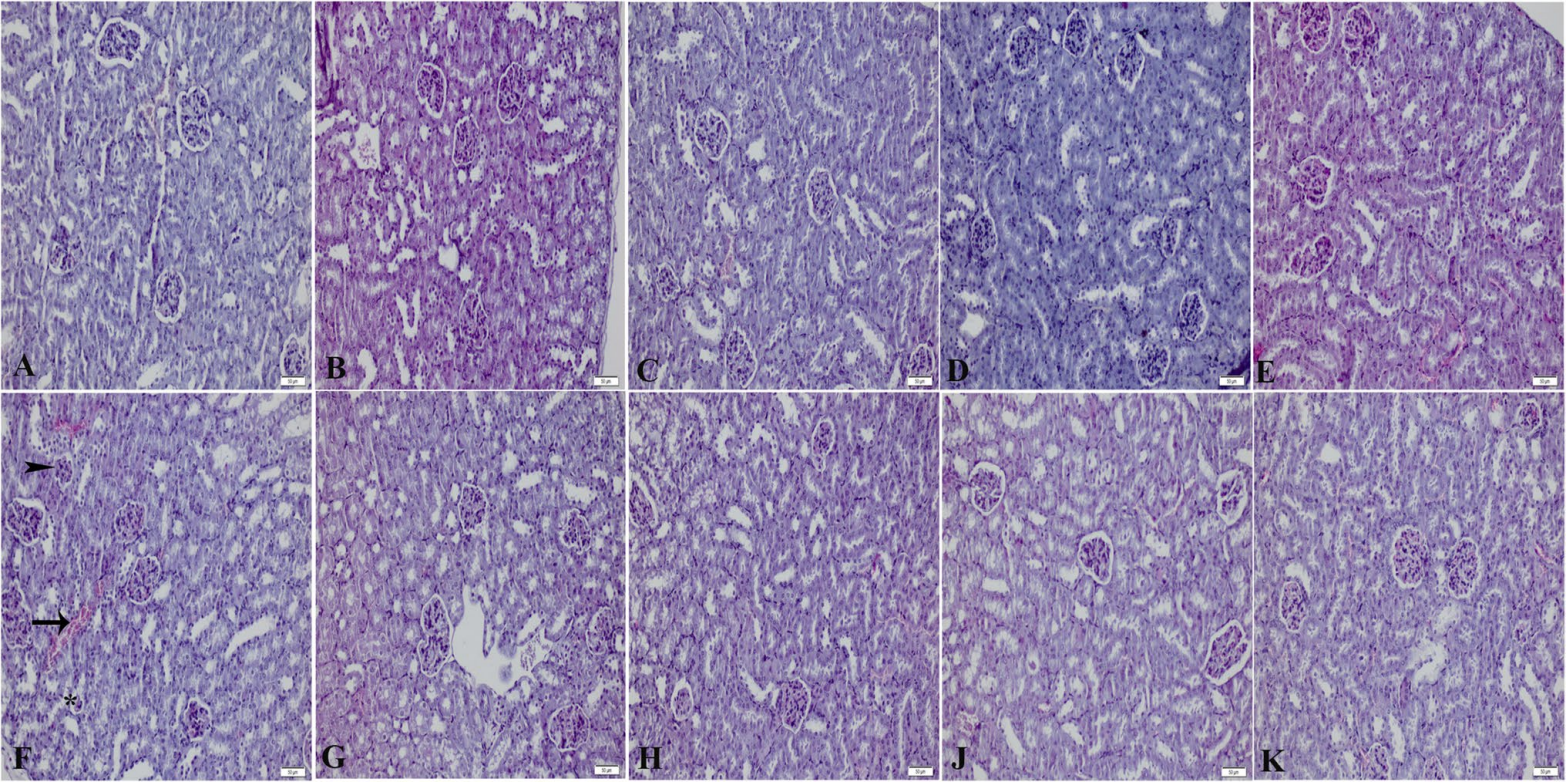
Renal tissue hematoxylin and eosin images. **A** CTRL group; **B** CTRL/DMSO group; **C** CTRL/LOS10 group; **D** CTRL/GW9662 group; **E** CTRL/LOS10/GW9662 group; **F** APAP group (arrow indicates hemorrhagic areas, arrowhead indicates glomerular damage, asterisk (*) indicates tubular epithelial shedding); **G** APAP/LOS1 group; **H** APAP/LOS3 group; **J** APAP/LOS10 group; and **K** indicates APAP/LOS10/GW9662 group. Image magnification at 200×

Renal tissue staining images are shown in Fig. 2. Normal histological images were obtained in the kidney tissues of the CTRL, CTRL/DMSO, CTRL/LOS10, CTRL/GW9662, and CTRL/LOS10/GW9662 groups. Glomerular damage, tubular epithelial shedding, and hemorrhagic areas were observed in the kidney tissue of the APAP group. These damages were absent in the APAP/LOS1, APAP/LOS3, APAP/LOS10, and APAP/LOS10/GW9662 groups.

## Discussion

In this study, whether losartan reduces hepatotoxicity and nephrotoxicity induced by acetaminophen in mice and its possible mechanisms of action were investigated. Although losartan is widely used in the treatment of hypertension, its protective effect has also been shown in the damage caused by ischemia/reperfusion in tissues such as the liver, kidney, and ovary due to its anti-inflammatory and antioxidant effects (Yao et al. 2007; An et al. 2010; Koh et al. 2013; Silveira et al. 2013; Hortu et al. 2020). Acetaminophen is widely used as an analgesic and antipyretic; hepatotoxicity due to overdose is one of the important problems frequently encountered in clinics (Shi et al. 2019; Ahmad et al. 2019). The limited therapeutic approaches related to this issue have led to the intensification of studies to find new therapeutic approaches (Zhao et al. 2018). The hepatotoxicity model induced by acetaminophen in experimental animals has greatly contributed to the progress of these studies (Ahmad et al. 2019). In our study, an increase in serum AST and ALT due to acetaminophen hepatotoxicity and creatinine levels due to nephrotoxicity was observed in accordance with other literature (Murad et al. 2016; Zhang et al. 2017; Sohrabinezhad et al. 2019). When hepatocellular integrity is disrupted for various reasons, ALT and AST leak into the bloodstream and their levels rise in the blood (Sohrabinezhad et al. 2019). Acute renal tubular necrosis and impaired renal function due to acetaminophen nephrotoxicity are manifested by increased serum creatinine levels (Murad et al. 2016). Losartan dose-dependently improved ALT and AST levels. It can be said that the reason for this improvement is due to the restoration of hepatocellular integrity by losartan. Similarly, losartan’s dose-dependent lowering of creatinine level could be explained by acetaminophen-induced reversal of renal tubular necrosis.

In a study evaluating the protective effect of taurine in acetaminophen-induced nephrotoxicity, it was determined that taurine reversed the increase in MDA and GSSG levels and the decrease in GSH SOD, CAT, and GPx levels which are oxidative stress markers in the kidney tissue, after acetaminophen administration. It was stated that the improvement in these markers indicates the protective effect of taurine on acetaminophen-induced nephrotoxicity (Das et al. 2010). In our study, the decrease in oxidative stress markers GSH, GPx, and TAS in kidney tissue after acetaminophen injection was reversed by losartan dose-dependently. This data shows that losartan reduces the oxidative stress in the kidney tissue and allows the healing of the kidney tissue. Oxidative stress is accepted as the main mechanism in hepatotoxicity induced by acetaminophen. Substances that have a protective effect on the liver mostly form activities by reducing oxidative stress (Ahmad et al. 2019). In our study, the administration of high-dose acetaminophen caused an increase in TOS, GSSG, and MDA levels, while decreasing the levels of TAS and GSH, which are oxidative stress markers. Losartan administration caused an improvement in serum TAS, GSH, TOS, GSSG, and MDA levels depending on the dose. In studies evaluating the protective efficacy of *Tinospora cordifolia* and *Boerhavia diffusa* extracts, irbesartan, n-acetylcysteine, and taurine in hepatotoxicity induced by acetaminophen; it has been shown that these test substances cause an increase in GSH and TAS levels after acetaminophen injection and a decrease in TOS, GSSG, and MDA levels (Acharya and Lau-Cam 2010; Kaushik et al. 2017b; Yoshioka et al. 2018; Helal and Samra 2020). The improvement in TAS, GSH, TOS, GSSG, and MDA levels, which are oxidative stress markers, can be considered an indication that losartan has a protective and curative effect by reducing oxidative stress in hepatotoxicity induced by acetaminophen. In a study evaluating the effect of taxifolin on hepatotoxicity induced by acetaminophen, it was shown that taxifolin improved the levels of MDA, ROS, GSH, and GPx in liver tissue. The improvement in these values was accepted as an indicator of the hepatoprotective effect of taxifolin (Hu et al. 2019). Similarly, in our study, it can be said that the improvement in oxidative stress markers in liver tissue is related to the hepatoprotective effect of losartan.

In our study, it was observed that there was more improvement in the levels of oxidative stress markers in the group in which losartan and GW9662 (PPAR-γ antagonist) were given together. Koh et al. (2013) contrary to their studies, in which they stated that losartan ameliorates I/R-induced liver damage via PPAR-γ; the data obtained as a result of our study indicate that antagonizing PPAR-γ increases the curative effect of losartan. In addition, Seargent et al. (2004) in their studies, the PPARγ antagonist GW9662 has been shown to inhibit the growth of human breast tumor cells. In the same study, it was determined that although GW9662 prevented the PPARγ activation caused by rosiglitazone, it increased the anticancer effect of rosiglitazone. Wojtowicz et al. (2014), in their study, showed that GW9662 caused an increase in the apoptotic and neurotoxic effects of TBBPA, although it prevented the decrease in PPARγ protein level caused by tetrabromobisphenol A (TBBPA). Considering the data we obtained in our study and these two studies, it can be said that not only PPARγ is involved in the improvement of acetaminophen-induced hepatotoxicity and nephrotoxicity by losartan, but also plays a role in different mechanisms.

In liver and kidney cells, some substances (nuclear DNA fragments, mitochondrial DNA (mtDNA), heat-shock proteins, hyaluronic acid, etc.) released together with the damage caused by excessive doses of acetaminophen induce the transcription of proinflammatory cytokines. The release of proinflammatory cytokines causes an increased concentration of neutrophils and monocytes, exacerbating liver and kidney tissue damage. Accordingly, the inflammation that develops as a result of acetaminophen administration causes further aggravation of the damage (Das et al. 2010; Silveira et al. 2013; Hartmut et al. 2014). In a study evaluating the effect of losartan in rats with experimental arthritis, due to the anti-inflammatory effect of losartan, it has been determined that it reduces joint hypernociception and helps to normalize joint functions (Silveira et al. 2013). In our study, IL-1β, IL-6, IL-8, IL-10, IL-17, and TNF-α levels, which are markers of the inflammatory response, increased after acetaminophen administration. Losartan administered at different doses caused a dose-dependent decrease in IL-1β, IL-6, IL-8, IL-10, IL-17, and TNF-α levels. When these results are evaluated, it can be said that the anti-inflammatory effect of losartan contributes to the improvement in acetaminophen hepatotoxicity and nephrotoxicity.

Oxidative stress and inflammation are two fundamental processes that play a crucial role in the pathogenesis of many pathological conditions. The complex interplay between these two biological events can lead to significant consequences at both the cellular level and the overall health of the organism. Oxidative stress arises from an increased formation of free radicals and the inadequacy of antioxidant defense systems within cells. Oxidative damage to cell membranes, proteins, and nucleic acids affects a cascade of signaling pathways within the cell (Sies 2015). Inflammation, on the other hand, is the organism’s defense mechanism against pathogens or tissue damage, initiated through cytokines, chemokines, and other mediators. These molecular signaling pathways are critical points influenced by oxidative stress (Sies 2015). Oxidative stress can initiate inflammation by causing cellular damage. Conversely, the inflammatory response can influence oxidative stress by regulating antioxidant systems. This mutual interaction can lead to extensive biochemical changes within cells and tissues (Valko et al. 2007). Specifically, lipid peroxidation and imbalance in the antioxidant system can lead to damage to liver cells. This situation plays a significant role in the pathogenesis of liver diseases, especially cirrhosis and fatty liver disease (Arroyave-Ospina et al. 2021). Oxidative stress can cause damage to renal cells, associated with chronic kidney diseases, glomerulonephritis, and other kidney pathologies (Wu et al. 2018). In our study, the observed adverse changes in markers of oxidative stress and inflammatory response following acetaminophen administration were reversed with Losartan treatment. This reversal can be considered an indication of the antioxidant and anti-inflammatory efficacy of Losartan.

High doses of acetaminophen have been shown to cause pyknotic nuclei, necrotic cells, and vacuolization in the liver (Ahmad et al. 2019). In kidney tissue, it has been determined that high-dose acetaminophen causes renal tubular damage and necrosis (Das et al. 2010). The necrosis, inflammatory regions, and pyknotic nuclei were observed in the liver tissue in the group in which acetaminophen was administered alone. However, these pathologies were not observed in the groups in which acetaminophen was administered together with losartan. Hemorrhagic areas, glomerular damage, and tubular epithelial shedding were observed in the group in which acetaminophen was administered alone. The kidney tissue in the acetaminophen + losartan group was observed to be similar to that of the control group. The histopathological findings obtained in the study support our biochemical findings.

## Conclusion

In conclusion, in this study, it was determined that losartan produced a remarkable improvement in acetaminophen-induced hepatotoxicity and nephrotoxicity. Inhibition of the harmful effects of acetaminophen in the liver and kidney through the antioxidant and anti-inflammatory effects of losartan may play a role in the curative effect of losartan. Thus, losartan was found to be an alternative substance that could protect people from these harmful effects of acetaminophen. These findings suggest that losartan could be used as a potential therapeutic agent for acetaminophen-induced liver and kidney injury.

## Data Availability

All data generated or analyzed during this study are included in this published article.

## References

[CR1] Abbasi A, Mawani H, Pathan GN (2021). Hepatoprotective role of virgin coconut oil and neem extract in acetaminophen ınduced liver toxicity. Pakistan J Med Heal Sci.

[CR2] Abdel-Daim MM, Khalifa HA, Abushouk AI (2017). Diosmin attenuates methotrexate-ınduced hepatic, renal, and cardiac ınjury: a biochemical and histopathological study in mice. Oxid Med Cell Longev.

[CR3] Acharya M, Lau-Cam CA (2010). Comparison of the protective actions of N-acetylcysteine, hypotaurine and taurine against acetaminophen-induced hepatotoxicity in the rat. J Biomed Sci.

[CR4] Ahmad MM, Rezk NA, Fawzy A, Sabry M (2019). Protective effects of curcumin and silymarin against paracetamol induced hepatotoxicity in adult male albino rats. Gene.

[CR5] Akin AT, Öztürk E, Kaymak E (2021). Therapeutic effects of thymoquinone in doxorubicin-induced hepatotoxicity via oxidative stress, inflammation and apoptosis. Anat Histol Embryol.

[CR6] Al Humayed S, Al-Ani B, El Karib AO (2019). Suppression of acetaminophen-induced hepatocyte ultrastructural alterations in rats using a combination of resveratrol and quercetin. Ultrastruct Pathol.

[CR7] Al Kury LT, Dayyan F, Ali Shah F (2020). Ginkgo biloba extract protects against methotrexate-ınduced hepatotoxicity: a computational and pharmacological approach. Molecules.

[CR8] An J, Nakajima T, Kuba K, Kimura A (2010). Losartan inhibits LPS-induced inflammatory signaling through a PPARγ-dependent mechanism in human THP-1 macrophages. Hypertens Res.

[CR9] Arroyave-Ospina JC, Wu Z, Geng Y, Moshage H (2021). Role of oxidative stress in the pathogenesis of non-alcoholic fatty liver disease: ımplications for prevention and therapy. Antioxidants.

[CR10] Budnitz DS, Lovegrove MC, Crosby AE (2011). Emergency department visits for overdoses of acetaminophen-containing products. Am J Prev Med.

[CR11] Das J, Ghosh J, Manna P, Sil PC (2010). Taurine protects acetaminophen-induced oxidative damage in mice kidney through APAP urinary excretion and CYP2E1 inactivation. Toxicology.

[CR12] El-Sokkary GH, Cuzzocrea S, Reiter RJ (2007). Effect of chronic nicotine administration on the rat lung and liver: beneficial role of melatonin. Toxicology.

[CR13] Goyal SN, Bharti S, Bhatia J (2011). Telmisartan, a dual ARB/partial PPAR-γ agonist, protects myocardium from ischaemic reperfusion injury in experimental diabetes. Diabetes Obes Metab.

[CR14] Güvenç M, Cellat M, Gökçek İ (2020). Nobiletin attenuates acetaminophen-induced hepatorenal toxicity in rats. J Biochem Mol Toxicol.

[CR15] Hartmut J, Yuchao X, Mitchell RM (2014). Acetaminophen-induced liver ınjury: from animal models to humans. J Clin Transl Hepatol.

[CR16] Helal MG, Samra YA (2020). Irbesartan mitigates acute liver injury, oxidative stress, and apoptosis induced by acetaminophen in mice. J Biochem Mol Toxicol.

[CR17] Hortu I, Ilgen O, Sahin C (2020). Losartan ameliorates ovarian ischaemia/reperfusion injury in rats: an experimental study. J Obstet Gynaecol (Lahore).

[CR18] Hu C, Ye J, Zhao L (2019). 5,7,3′,4′-flavan-on-ol (taxifolin) protects against acetaminophen-induced liver injury by regulating the glutathione pathway. Life Sci.

[CR19] Kalantari H, Asadmasjedi N, Abyaz M, reza,  (2019). Protective effect of inulin on methotrexate-induced liver toxicity in mice. Biomed Pharmacother.

[CR20] Kaplowitz N (2005). Idiosyncratic drug hepatotoxicity. Nat Rev Drug Discov.

[CR21] Kaushik A, Husain A, Awasthi H (2017). Antioxidant and hepatoprotective potential of Swaras and Hima extracts of *Tinospora cordifolia* and *Boerhavia diffusa* in Swiss albino mice. Pharmacogn Mag.

[CR22] Kaushik A, Husain A, Awasthi H (2017). Antioxidant and hepatoprotective potential of swaras and hima extracts of *Tinospora cordifolia* and *Boerhavia diffusa* in Swiss albino mice. Pharmacogn Mag.

[CR23] Kaymak E, Öztürk E, Akin AT (2022). Thymoquinone alleviates doxorubicin induced acute kidney injury by decreasing endoplasmic reticulum stress, inflammation and apoptosis. Biotech Histochem.

[CR24] Koh EJ, Yoon SJ, Lee SM (2013). Losartan protects liver against ischaemia/reperfusion injury through PPAR-γ activation and receptor for advanced glycation end-products down-regulation. Br J Pharmacol.

[CR25] Larson AM (2007). Acetaminophen hepatotoxicity. Clin Liver Dis.

[CR26] McGill MR, Lebofsky M, Norris HRK (2013). Plasma and liver acetaminophen-protein adduct levels in mice after acetaminophen treatment: dose–response, mechanisms, and clinical implications. Toxicol Appl Pharmacol.

[CR27] Mitchell JR, Jollow DJ, Potter WZ (1973). Acetaminophen-induced hepatic necrosis. IV. Protective role of glutathione. J Pharmacol Exp Ther.

[CR28] Murad HAS, Habib H, Kamel Y (2016). Thearubigins protect against acetaminophen-induced hepatic and renal injury in mice: biochemical, histopathological, immunohistochemical, and flow cytometry study thearubigins protect against acetaminophen-induced hepatic and. Drug Chem Toxicol.

[CR29] Pang T, Benicky J, Wang J (2012). Telmisartan ameliorates lipopolysaccharide-induced innate immune response through peroxisome proliferator-activated receptor-γ activation in human monocytes. J Hypertens.

[CR30] Seargent JM, Yates EA, Gill JH (2004). GW9662, a potent antagonist of PPARγ, inhibits growth of breast tumour cells and promotes the anticancer effects of the PPARc agonist rosiglitazone, independently of PPARγ activation. Br J Pharmacol.

[CR31] Shi C, Hao B, Yang Y (2019). JNK signaling pathway mediates acetaminophen-induced hepatotoxicity accompanied by changes of glutathione S-transferase A1 content and expression. Front Pharmacol.

[CR32] Sies H (2015). Oxidative stress: a concept in redox biology and medicine. Redox Biol.

[CR33] Silveira KD, Coelho FM, Vieira AT (2013). Mechanisms of the anti-inflammatory actions of the angiotensin type 1 receptor antagonist losartan in experimental models of arthritis. Peptides.

[CR34] Sohrabinezhad Z, Dastan D, Asl SS, Nili-Ah-Madabadi A (2019). Allium jesdianum extract ımprove acetaminophen-ınduced hepatic failure through ınhibition of oxidative/nitrosative stress. J Pharmacopuncture.

[CR35] Uetrecht J, Naisbitt DJ (2013). Idiosyncratic adverse drug reactions: current concepts. Pharmacol Rev.

[CR36] Ulusoy HB, Öztürk İ, Sönmez MF (2016). Protective effect of propolis on methotrexate-induced kidney injury in the rat. Ren Fail.

[CR37] Valko M, Leibfritz D, Moncol J (2007). Free radicals and antioxidants in normal physiological functions and human disease. Int J Biochem Cell Biol.

[CR38] Wojtowicz AK, Szychowski KA, Kajta M (2014). PPAR-γ agonist GW1929 but not antagonist GW9662 reduces TBBPA-induced neurotoxicity in primary neocortical cells. Neurotox Res.

[CR39] Wu Q, Wang X, Nepovimova E (2018). Mechanism of cyclosporine A nephrotoxicity: oxidative stress, autophagy, and signalings. Food Chem Toxicol.

[CR40] Yao EH, Fukuda N, Matsumoto T (2007). Losartan improves the impaired function of endothelial progenitor cells in hypertension via an antioxidant effect. Hypertens Res.

[CR41] Yılmaz S, Tokpınar A, Ateş Ş (2020). Effect of *Cornus mas* l. Extract on organs in rats given nicotine. New Trends Med Sci.

[CR42] Yoshioka H, Nonogaki T, Ohnishi H (2018). 1O, 20O-diacetyl kamebakaurin protects against acetaminophen-induced hepatotoxicity in mice. Biomed Res.

[CR43] Zaher H, Buters JTM, Ward JM (1998). Protection against acetaminophen toxicity in CYP1A2 and CYP2E1 double-null mice. Toxicol Appl Pharmacol.

[CR44] Zhang T-L, Fu J-L, Geng Z (2012). The neuroprotective effect of losartan through inhibiting AT1/ ASK1/MKK4/JNK3 pathway following cerebral I/R in Rat hippocampal CA1 region. CNS Neurosci Ther.

[CR45] Zhang J, Song Q, Han X (2017). Multi-targeted protection of acetaminophen-induced hepatotoxicity in mice by tannic acid. Int Immunopharmacol.

[CR46] Zhao Z, Wei Q, Hua W (2018). Hepatoprotective effects of berberine on acetaminophen-induced hepatotoxicity in mice. Biomed Pharmacother.

